# Potentialities of High-Resolution 3-D CZT Drift Strip Detectors for Prompt Gamma-Ray Measurements in BNCT

**DOI:** 10.3390/s22041502

**Published:** 2022-02-15

**Authors:** Leonardo Abbene, Fabio Principato, Antonino Buttacavoli, Gaetano Gerardi, Manuele Bettelli, Andrea Zappettini, Saverio Altieri, Natalia Auricchio, Ezio Caroli, Silvia Zanettini, Nicoletta Protti

**Affiliations:** 1 Department of Physics and Chemistry (DiFC)—Emilio Segrè, University of Palermo, Viale delle Scienze, Edificio 18, 90128 Palermo, Italy; leonardo.abbene@unipa.it (L.A.); antonino.buttacavoli@unipa.it (A.B.); gaetano.gerardi@unipa.it (G.G.); 2 IMEM/CNR, Parco Area delle Scienze 37/A, 43100 Parma, Italy; manuele.bettelli@imem.cnr.it (M.B.); andrea.zappettini@imem.cnr.it (A.Z.); 3 Department of Physics, University of Pavia and Nuclear Physics National Institute (INFN), Unit of Pavia, Via Agostino Bassi 6, 27100 Pavia, Italy; saverio.altieri@unipv.it (S.A.); nicoletta.protti@unipv.it (N.P.); 4 INAF/OAS Bologna, 40129 Bologna, Italy; natalia.auricchio@inaf.it (N.A.); ezio.caroli@inaf.it (E.C.); 5 due2lab s.r.l., Via Paolo Borsellino 2, 42019 Scandiano, Italy; zanettini@due2lab.com

**Keywords:** CZT detectors, X-ray and gamma-ray detectors, BNCT

## Abstract

Recently, new high-resolution cadmium–zinc–telluride (CZT) drift strip detectors for room temperature gamma-ray spectroscopic imaging were developed by our group. The CZT detectors equipped with orthogonal anode/cathode collecting strips, drift strips and dedicated pulse processing allow a detection area of 6 × 20 mm^2^ and excellent room temperature spectroscopic performance (0.82% FWHM at 661.7 keV). In this work, we investigated the potentialities of these detectors for prompt gamma-ray spectroscopy (PGS) in boron neutron capture therapy (BNCT). The detectors, exploiting the measurement of the 478 keV prompt gamma rays emitted by 94% ^7^Li nuclides from the ^10^B(n, α)^7^Li reaction, are very appealing for the development of single-photon emission computed tomography (SPECT) systems and Compton cameras in BNCT. High-resolution gamma-ray spectra from ^10^B samples under thermal neutrons were measured at the T.R.I.G.A. Mark II research nuclear reactor of the University of Pavia (Italy).

## 1. Introduction

Boron neutron capture therapy (BNCT) is a radiation therapy exploiting the high probability of ^10^B to capture thermal neutrons through the nuclear reaction ^10^B(n, α)^7^Li. The products of this reaction (^7^Li nuclides and alpha particles) are characterized by high linear energy transfer (LET), of about 150 keV/μm for alpha particles and 175 keV/μm for ^7^Li nuclei, with important potentialities in destroying cancer cells. The idea of BNCT was first proposed by Gordon Locher in 1936 [1], the first research activities were pioneered by Kruger in 1940 [2] and the clinical tests started in the USA in 1951 [3]. Since then, several clinical studies have been made around the world—the pioneering works in Japan [4], Argentina [5], Finland [6], Sweden [7] and Italy [8]. The optimization of the treatment effects in BNCT requires the knowledge of the real distribution of ^10^B concentration in both tumor and healthy cells, possibly during the irradiation. For real-time measurements of the ^10^B distribution, the prompt gamma-ray spectroscopy (PGS) technique [9,10] is considered the most appealing procedure. The key operation is the direct measurement of the 478 keV prompt gamma rays emitted by 94% ^7^Li nuclides from the ^10^B(n, α)^7^Li reaction. Conventional PGS instrumentation is based on single photon emission computed tomography (SPECT) systems, equipped with the proper energy resolution to effectively detect the 478 keV gamma rays from the gamma-ray background created by various (n, γ) reactions and annihilation phenomena. Typical SPECT systems are based on scintillators [11] or semiconductor detectors [12] matched to dedicated mechanical collimators for image reconstruction. Intense activities have been made on SPECT systems based on high-Z and wide-bandgap compound semiconductors [12,13] in order to ensure room temperature operation (if compared with the cryogenically cooled germanium detectors), high detection efficiency and energy resolution better than scintillators. Among these, cadmium telluride (CdTe) and cadmium–zinc–telluride (CdZnTe or CZT) detectors showed interesting room temperature performance for BNCT-SPECT systems, with an energy resolution of 2–3% at 662 keV [14,15,16,17,18,19]. High-resolution BNCT-SPECT prototypes (<2% at 662 keV), based on CdTe Schottky detectors, have been developed [12,15]; however, to minimize the time instability due to the bias-induced polarization [20,21], the thickness of CdTe Schottky detectors must be less than 2 mm, thus limiting the detection efficiency and the detection area. CdTe detectors with ohmic contacts allow greater thickness but poorer energy resolution (>2% at 662 keV) [14]. CZT detectors with ohmic contacts, due to their higher resistivity than the CdTe ones, should ensure better room temperature energy resolution; but the presence of more charge trapping and low crystal uniformity requires more effort in both pulse processing and crystal growth. BNCT-SPECT CZT prototypes with standard pulse processing electronics showed energy resolution >2% at 662 keV [17,18,19]. In order to reduce the limits of the mechanical collimators in BNCT-SPECT systems, in terms of sensitivity and spatial resolution, Compton cameras were also investigated [22,23,24]. This approach, based on the analysis of the kinematics of the Compton scattering, requires scatter/absorber spectrometers with 3-D positioning and timing capabilities.

Recently, in the framework of the 3CaTS project (funded by INFN, Italy), new high-resolution 3-D CZT drift strip detectors were developed for prompt gamma-ray spectroscopy in BNCT. The CZT prototypes are characterized by a detection area of 6 × 20 mm^2^ and equipped with custom 32-channel digital electronics able to detect and analyze single and double events from Compton/photoelectric photon interactions. The goal of the project was to develop new CZT detectors able to ensure room temperature energy resolution <2% at 662 keV and 3-D spatial resolution < 1 mm, as prototypes for both BNCT-SPECT and BNCT-Compton cameras.

In this work, we present, besides the spectroscopic capabilities of the detectors, the first results from a ^10^B-enriched sample irradiated by the highly thermalized neutron beam of the Prompt Gamma Neutron Activation Analysis (PGNAA) facility at the T.R.I.G.A. Mark II research nuclear reactor of the University of Pavia (Pavia, Italy).

## 2. The 3-D CZT Drift Strip Detectors

**Figure 1:**
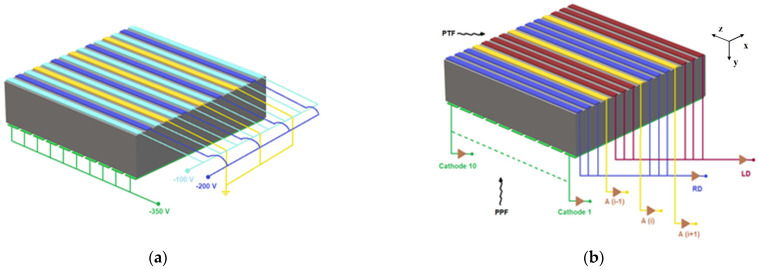
(**a**) Electrical scheme of the bias voltage setting for the anode the cathode strips. (**b**) The signal read−out setting and the irradiation geometries: planar parallel field (PPF) and planar transverse field (PTF). The PTF geometry allows a detection area of 6 × 20 mm^2^ (X–Y plane) and a photon interaction depth of 20 mm.

The first CZT detectors for X-ray and gamma-ray spectroscopy were developed in 1992 [25] and their capabilities in detecting thermal neutrons were first presented in 1996 [26]. Since then, great efforts and successful results have been obtained in the development of high-resolution CZT detectors, in terms of both crystal growth [27,28] and device/electrode technology [29,30,31,32]. CZT detectors with orthogonal anode and cathode strips were fabricated since 1996 [33] and important advances were obtained at the DTU Space (Denmark) for astrophysical applications [34,35]. The strip electrode layout and the drift strips allow charge collection enhancements and sub-millimeter 3-D spatial capabilities. Recently, new CZT drift strip detectors with excellent room temperature performance were developed by our group [36,37]. The detectors, based on CZT crystals (19.4 × 19.4 × 6 mm^3^) grown by the traveling heather method (THM) technique [28], were fabricated at IMEM-CNR of Parma (Italy) with the collaboration of the due2lab company (Scandiano, Italy) [36,37]. In particular, the detectors were developed from THM-CZT pixel detectors (Redlen Technologies, Canada) with enhanced electron charge transport properties (μ_e_τ_e_ > 10^−2^ cm^2^/V). The strips were realized with gold electro-less contacts with a deposition of thin Al_2_O_3_ film between them, ensuring very low leakage currents (<1 nA at 50 V between the strips) [38]. The gold strip electrodes were deposited from solution [39] and cathode and anode patterning were performed in two different steps. The electrode configuration is based on orthogonal strips on the anode and cathode sides: the anode strips have a pitch of 0.4 mm (strip width of 0.15 mm and inter-strip gap of 0.25 mm) (Figure 1), whereas the cathode strips are orthogonal to the anode strips with a pitch of 2 mm (strip with of 1.9 mm and inter-strip gap of 0.1 mm). To optimize the electron charge collection on the collecting strips (the yellow strips of Figure 1), the adjacent strips, termed drift strips, are negatively biased, as clearly shown in Figure 1a. In particular, the drift strips (light blue) more adjacent to the collecting strips are biased at −100 V and the central drift strips (blue) at −200 V; the cathodes are biased at −350 V. The signal read-out of the strips is organized as follows (Figure 1b): the collecting anode (yellow) and the cathode (green) strips are singly read out, while the drift strips are combined together in two groups of three strips; the drift strips (red) on the right side (RD) and the strips (violet) on the left side (LD) of each collecting strip.

The detectors are divided into 12 functional strip groups, termed drift cells. Each drift cell is characterized by a collection anode, the RD and LD strips and the 10 cathode strips. Each drift cell is able to provide both the energy and the 3-D interaction position of the photons, as already demonstrated in the literature [34,35]. In particular, the energy of the photons can be given by the pulses from the collecting anodes, the x-position by the collecting anodes related to the RD/LD anode pulses, the y-position through the relation between the cathode and the anode pulses and the z-position by the cathode pulses. Despite a pitch of 1.6 mm of the drift cells and 2 mm of the cathode strips, it is possible to reduce the spatial resolution through the analysis of the pulses from the drift strips [34,35].

**Figure 2:**
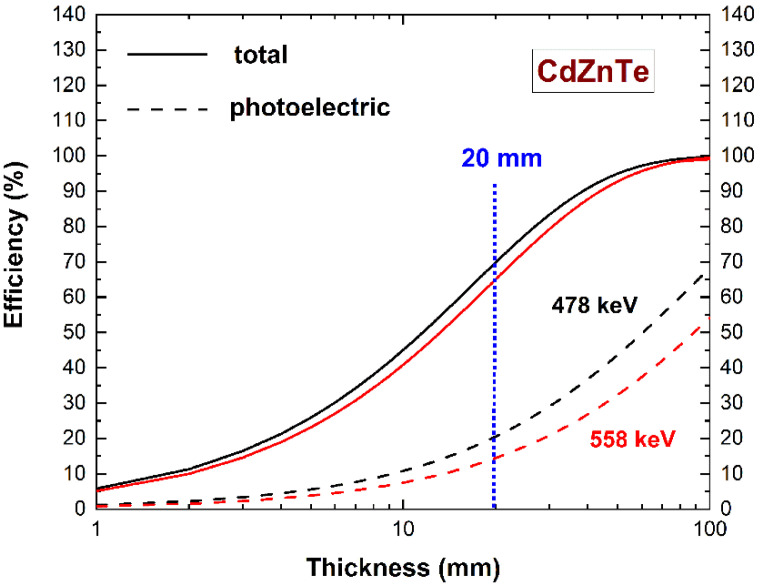
Calculated detection efficiency versus CZT material thickness. The energies are the typical gamma energies of the ^10^B(n, α )^7^Li and ^113^Cd(n, γ )^114^Cd reactions.

In order to exploit the 20 mm thickness of CZT material over the z-direction (Figure 1b), the detectors were developed to work in the planar transverse field (PTF) irradiation geometry (i.e., photons interacting on the X–Y plane). The energies of 478 keV and 558 keV, shown in Figure 2, are the main prompt gamma-rays emitted by the ^10^B(n, α)^7^Li and ^113^Cd(n, γ)^114^Cd reactions, respectively.

The detector strips were connected to low-noise (equivalent noise charge ENC of 100 electrons) charge-sensitive preamplifiers (CSPs) and processed by 32-channel digital electronics. Both the CSPs and the digital electronics were developed at the University of Palermo (Palermo, Italy) [36,40,41]. The digital electronics allows the application, for each CSP pulse, of fast (for event detection) and slow shaping (for energy measurements) for the proper detection and analysis of single photoelectric and double Compton/photoelectric events.

### 2.1. The Pulses from the Strips: Single and Double Photon Interactions

**Figure 3:**
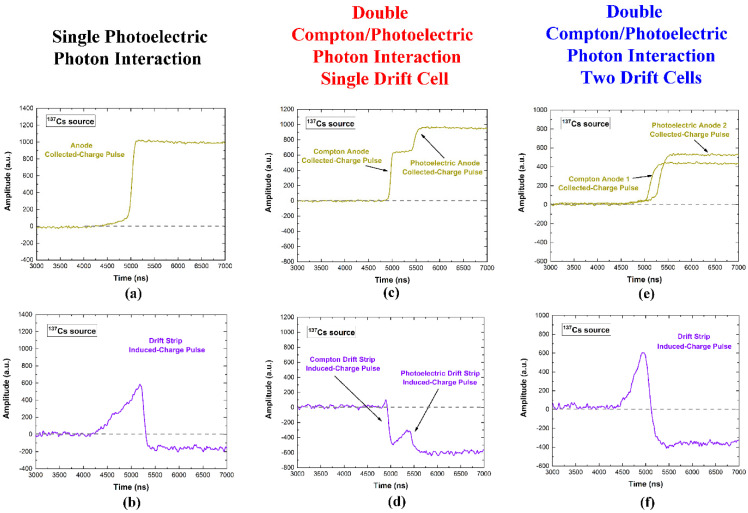
Examples of single and double interaction pulses from the collecting anodes and the related drift strips, measured in temporal coincidence. (**a**) Single photoelectric collected-charge pulse from a collecting anode and (**b**) the related induced-charge pulse from the drift strip. (**c**) Compton and photoelectric collected-charge pulses from one collecting anode and (**d**) the related induced-charge pulses from the drift strip; both pulses are detected within a single drift cell. (**e**) Compton and photoelectric collected-charge pulses from two collecting anodes and (**f**) the related induced-charge pulses from the drift strip; here, the pulses are detected by two different drift cells.

**Figure 4:**
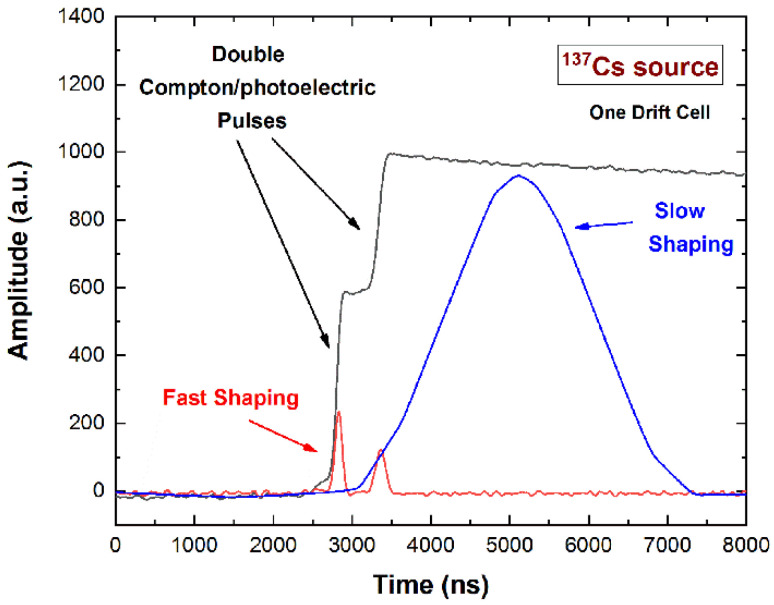
Double pulses (black line) generated on a single drift cell from Compton/photoelectric interactions. The shaped pulse (blue line) from a standard slow shaping (pulse time with of about 4 μs), optimized and used to analyze single photoelectric pulses, gives rise to a false single pulse. While, the shaped pulses (red line) from a fast shaping (pulse time with of about 200 ns) really highlight the presence of the double pulses. The digital electronics allow, for each detector pulse, the analysis with both fast and slow shaping, implemented in pipeline.

As well described in our previous work [36], the collecting anode strips mainly give rise to collected-charge pulses, i.e., pulses due to the electrons really collected by the strips; while induced-charge pulses characterize the drift strips: they are induced by the motion of the electrons really collected by the adjacent collecting anode strips. The shape of the pulses strongly depends on the photon interaction position and it is mainly related to the particular behavior of the weighting potential and charge trapping of electrons and holes. The details of the characteristics of these pulses are better described in our previous work [36]. Here, to highlight the potentialities of the detectors as Compton cameras, we focus on the shape of the pulses from the collecting anodes and the adjacent drift strips, due to single photoelectric or double interaction by Compton/photoelectric events. In Figure 3, the single output CSP pulses from photoelectric interaction, the double pulses from Compton/photoelectric interactions within a single drift cell and within two drift cells are presented. The detector, working as both scatter and absorber, is able to detect double events from Compton/photoelectric interaction even within a single drift cell. Generally, the double events within a single drift cell are not well detected by the standard pulse processing electronics; the detection of these events strongly increases the sensitivity of Compton cameras. In fact, as shown in Figure 4, the double events from Compton/photoelectric interactions within a single drift cell are detected as false single events by a standard pulse processing; therefore, they are wrongly detected and analyzed as single photoelectric events. In fact, the slow shaping (blue line), optimized to analyze the single photoelectric events, gives rise to a single pulse. While, through the fast shaping (red line), it is possible to correctly detect the double events. This operation is allowed by our digital electronics that perform on each CSP pulse both fast and slow shaping processing. As already demonstrated in our previous work [36], the negative saturation levels of the induced pulses from the drift strips are due to the trapping of the holes. It is possible to take into account this saturation level for improvements in energy resolution. The presence of double events is also visible in the induced pulses from the drift strips (Figure 3d).

### 2.2. Spectroscopic Performance

**Figure 5:**
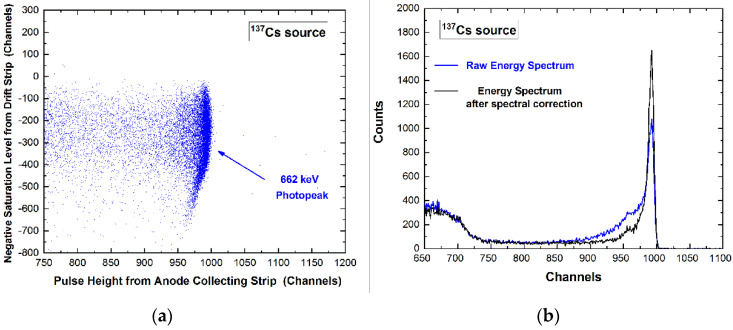
(**a**) Two-dimensional (2D) scatter plot of the negative saturation level of the pulses from a drift strip versus the height of pulses from an anode collecting strip. The curvature highlights the presence of pulses with incomplete charge collection related to high values of the negative saturation level. (**b**) Measured ^137^Cs energy spectra after the spectral correction (black line) [36]. The events in the tailing of the photopeak, i.e., characterized by incomplete charge collection, are well detected and corrected.

The detectors show interesting room temperature spectroscopic performance even without the application of spectral correction techniques; in particular, the raw energy spectra (i.e., with no spectral correction) are characterized by energy resolution FWHM of 5.0% and 1.3% at 122.1 keV and 661.7 keV, respectively. As reported in our previous work [36], it is possible to improve the energy resolution through the relation between the height (i.e., the energy) of the pulses of the investigated collecting strip with some features of the pulses from the cathode and drift strips. For example, it is possible to use the height or the peaking time of the cathode pulses, the peaking time of the collecting anode pulses and the negative saturation level of the pulses from the drift strips. The best results are obtained by using the negative saturation levels from the drift strip pulses, as shown in Figure 5.

**Figure 6:**
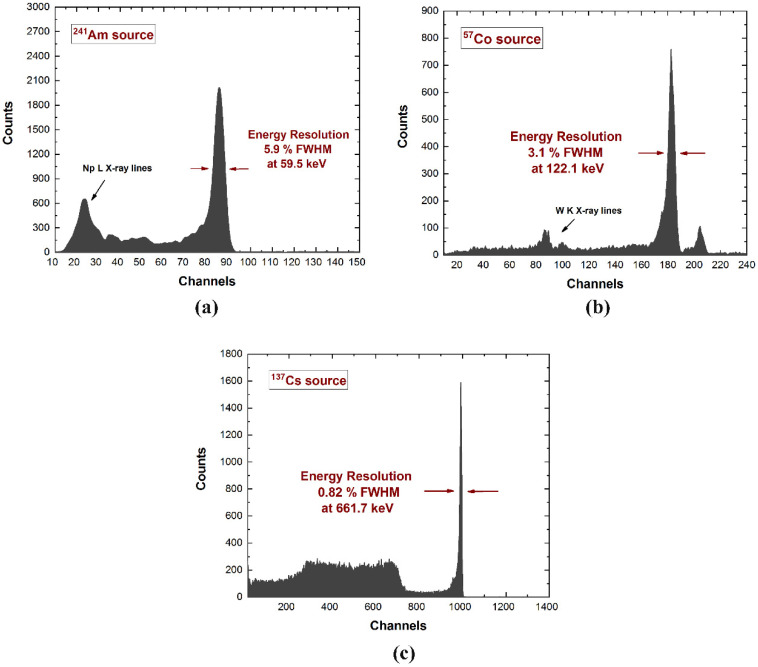
Uncollimated (**a**) ^241^Am, (**b**) ^57^Co and (**c**) ^137^Cs energy spectra measured with the CZT drift strip detectors. The excellent energy resolution was obtained after the application of a novel correction technique using the negative saturation levels from the drift strips [36].

Figure 6 shows the energy spectra from uncollimated radiation sources (^241^Am, ^57^Co, ^137^Cs), measured through a collecting anode after the spectral correction with the negative saturation levels from the drift strips [36]. The detectors are characterized by excellent energy resolution at room temperature (5.9% FWHM at 59.5 keV; 3.1% FWHM at 122.1 keV; 0.82% FWHM at 661.7 keV).

## 3. Prompt Gamma-Ray Measurements in BNCT Environment

The gamma-ray response of the detectors to ^10^B signal was investigated at the T.R.I.G.A. Mark II research nuclear reactor of the University of Pavia (Pavia, Italy). The T.R.I.G.A. Mark II reactor is a water-cooled and water-moderated reactor, equipped with several in-core and out-core irradiation channels and positions, allowing a flux along the core axis of 5 × 10^13^ cm^−2^ s^−1^ at the full power (250 kW). To measure the typical gamma-ray products from the ^10^B neutron capture reaction leading to the BNCT therapeutic effects, a small liquid sample containing ^10^B was irradiated with a highly thermalized neutron beam of the Prompt Gamma Neutron Activation Analysis (PGNAA) facility recently installed at the reactor. In particular, we used almost 1 mL liquid sample containing ^10^B at a concentration of approximately 5000 ppm and irradiated at the reactor power of 20 kW, corresponding to a thermal neutron flux of almost 5.3 × 10^5^ cm^−2^ s^−1^ at the PGNAA beam port.

The high ^10^B concentration of the sample is explained as follows: at the maximum reactor power of 250 kW, the thermal neutron flux at the PGNAA beam port is about 5∙× 10^6^ n/cm^2^s, thus almost 3-2 orders of magnitude less than what expected in a BNCT clinical scenario. To balance this loss and still achieve the reaction rate of ^10^B expected in the clinical application, we have been forced to increase the ^10^B concentration. The self-shielding effect at this level of ^10^B inside the sample is certainly high (preliminary estimations around 45%).

**Figure 7:**
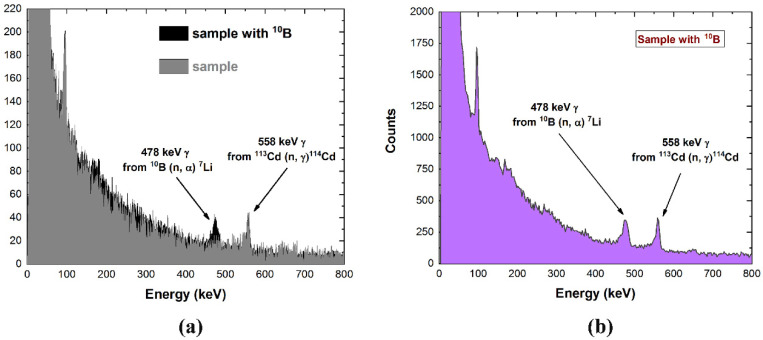
Gamma-ray energy spectra measured with the CZT drift strip detectors in a BNCT environment. (**a**) A comparison between the presence (black line) and the absence of ^10^B (gray line). (**b**) The measured energy spectrum with higher counting statistics. The flat top of the 478 keV peak highlights the Doppler broadening effect. We used the spectral correction described above.

The CZT drift strip detector was positioned orthogonally to the irradiation direction, in PTF geometry and without collimators. The expected measured gamma rays should be mainly represented by: (a) the 2223 keV gamma rays from the ^1^H(n,γ)^2^H reaction and the related Compton scattered photons, (b) the annihilation gamma-rays (mainly due to 2223 keV photon interaction on shielding/collimation materials), (c) various (n, γ) reaction products coming from the neutron activation of the walls and materials composing the PGNAA facility and (d) the desired 478 keV gamma rays from the ^10^B(n, α)^7^Li reaction. In CZT detectors, the 558 keV and 651 keV gamma rays from ^113^Cd(n, γ)^114^Cd neutron capture reaction are also typically measured [26]. Figure 7 shows the measured energy spectra from ^10^B under the described conditions. In particular (Figure 7a), the energy spectra with the presence and absence of ^10^B, simulating tumor, tissues and background, are presented.

A photon-counting rate of 2.4 cps for the 478 keV gamma rays was measured. Measurements with higher counting statistics are shown in Figure 7b. The 478 keV and the 558 keV peaks are well detected and resolved, with important spectral improvements with respect to our previous results obtained with other CZT detector prototypes [18]. The high energy resolution of the detectors also allows detecting the expected different shapes between the 478 keV and the 558 keV peaks due to the Doppler effect, since the prompt gamma rays are emitted in flight by ^7^Li. In fact, the flat top of the 478 keV peak clearly highlights the effects of the Doppler broadening. To our knowledge, this is the first measurement, performed with room temperature CdTe/CZT detectors, clearly highlighting this effect; generally, these different peak shapes are masked by the poor energy resolution of the detectors and they are typically measured with high-resolution cryogenic cooled germanium detectors [14]. These measurements confirm the capabilities of these new CZT detectors to discriminate the ^10^B signal properly in BNCT despite the close proximity of the activation peak of ^113^Cd. The absence of shielding/collimation materials did not allow us to experimentally ascertain the separation of the ^10^B peak from the annihilation one. Nonetheless, the extremely good energy resolution performances of the detectors strongly support this conclusion.

## 4. Conclusions

The spectroscopic potentialities of new CZT drift strip detectors for SPECT systems and Compton cameras in BNCT are presented. The detectors, due to the application of original spectral correction techniques, are characterized by excellent room temperature energy resolution, about 3% FWHM at 122 keV and 0.8% FWHM at 662 keV. The detection of both single photoelectric events and double Compton/photoelectric events allows the detectors to work as scatter/absorber detectors for Compton cameras. In particular, the digital electronics, through the application of both fast and slow shaping, allows the detection of double Compton/photoelectric events within a single detection cell, with important enhancements in terms of sensitivity for Compton cameras. Measurements under thermal neutrons highlighted the abilities of the detectors to well detect the 478 keV photons from the parasitic neutron activation peaks of ^113^Cd. The effects of the Doppler broadening on the 478 keV peak, typically detected with cryogenic cooled germanium detectors, are clearly visible. Ongoing activities are focused on the spatial characterization of the detectors, by exploiting charge sharing and induced signals, for image reconstruction of the ^10^B distribution during a BNCT treatment, working as SPECT and Compton camera.
